# Consumers' attention on identification, nutritional compounds, and safety in heavy metals of Canadian sea cucumber in Chinese food market

**DOI:** 10.1002/fsn3.1882

**Published:** 2020-09-13

**Authors:** Zhuoyue Song, Hailun Li, Jing Wen, Yeda Zeng, Xianying Ye, Weibo Zhao, Tingting Xu, Nenggui Xu, Danyan Zhang

**Affiliations:** ^1^ Clinical Medical College of Acupuncture Moxibustion and Rehabilitation School of Pharmaceutical Science Guangzhou University of Chinese Medicine Guangzhou China; ^2^ Department of Nephrology Affiliated Huai'an Hospital of Xuzhou Medical University Huai'an China; ^3^ Department of Biology Lingnan Normal University Zhanjiang China; ^4^ Jiangsu Key Laboratory of Regional Resource Exploitation and Medicinal Research Huaiyin Institute of Technology Huai'an China

**Keywords:** active ingredients, *Cucumaria frondosa*, identification, safety in heavy metal, sea cucumber

## Abstract

**Background:**

Based on the consumers’ attention issues of sea cucumbers, we aimed to complete comprehensive information of commercial Canadian sea cucumbers (CCSC), which sprang up extensively in Chinese food market.

**Results:**

CCSC were identified as *Cucumaria frondosa* and characterized based on the characteristics, nutritional compositions, and heavy metals. The abdomen and five internal tendons of *Cucumaria frondosa* were special orange. The average of soaking degree and water content, which consumers paid great attention to, was 2.8 ± 0.3 and 0.46 ± 0.09%, respectively. Proteins (56.4 ± 9.1%) and polysaccharides (12.2 ± 14.7%) were the principal nutrient component. In addition, there was a variety of free amino acids, in which arginine (70.1 ± 50.0 mg/100 g), glutamate (42.6 ± 23.9 mg/100 g), and alanine (32.2 ± 21.0 mg/100 g) were the main components. Phosphorus (P, 0.26 ± 0.05%), magnesium (Mg, 0.19 ± 0.07%), and kalium (K, 0.17 ± 0.08%) were the major mineral elements. Amount of heavy metal was within the safety limitation (5.5 ± 1.4 mg/kg). Furthermore, the active ingredients were positively correlated with size.

**Conclusion:**

The overall findings enriched the information of *Cucumaria frondosa* for consumers and suggested that the quality of *Cucumaria frondosa* was varied following commercial classification and size.

## Graphical abstract

This paper would enrich the information of *Cucumaria frondosa* for consumers and suggest that the quality of *Cucumaria frondosa* was varied following commercial classification and size.

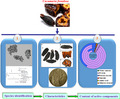

## 1 INTRODUCTION

Sea cucumbers, also called bêche‐de‐mer, belong to the class Holothuroidea (phylum Echinodermata), which are usually processed into dried product and valued as important seafood delicacies particularly in China, Japan, and South Asia (Correia‐da‐Silva, Sousa, Pinto, & Kijjoa, 2017). Based on nutritional view, sea cucumbers are considered to be ideal tonics due to their profile of high‐valued nutrients such as protein, polysaccharides, saponins, amino acids, and mineral elements (Bordbar, Anwar, & Saari, 2011; Silchenko et al., 2005), which possess the effect of antiviral, antibacterial, anti‐inflammatory, anti‐aging, regulation of blood sugar and blood pressure, anti‐angiogenesis, immunomodulation, and anti‐tumor (Aminin et al., 2008; Wang, Han, Chen, Yi, & Sun, 2014). Moreover, sea cucumbers, as traditional foods and folk medicine in many areas of the world, have gained immense popularity and interest due to their nutritive value and potential health benefits.

Based on the concerned issues of consumers in choice and purchase of sea cucumbers, a questionnaire was prepared. A total of 686 people participated in the questionnaire who bought or interested in sea cucumbers. And among the consumers, women accounted for 58.16% and men accounted for 41.84%. The age of the consumers was mainly concentrated between 20 and 50 years old, accounting for 87.9% of the total number. Nutritional components, price, safety, activities, scope of application, taste, usage and dosage, locality, size and specification, variety, brand, processing methods and others were prepared for consumers to choose 3–5 more important options. According to the results of market research reports (Figure S1), which exceed 50%, consumers paid more attention to was price (70.55%), nutritional components (68.08%), and safety (65.01%).

Nowadays, many species of sea cucumber are being cultured and fished to meet the high demand of great global production and trade (Chen, 2003). However, wild sea cucumber resources have been depleted and the production of sea cucumber products heavily relies upon the aquaculture culture of sea cucumbers (Nelson, MacDonald, & Robinson, 2012). Despite the improved culture techniques, demand for sea cucumber products is still increasing, thus the importation of additional non‐native species is necessary. Canadian sea cucumber is a naturally nutritious food mainly from Canada and labeled with the local denomination in China. The Canadian sea cucumbers, which were considered cheap, wild and with good quality, extensively sprang up in the Chinese food market in recent years. The lower price compared with other sea cucumber mostly may be related to that Canadian sea cucumber is a non‐native species and has not been recognized by Chinese consumers. Although 82.44% of consumers did not know Canadian sea cucumber in the survey, 67.77% were interested in it. However, the species, characteristics, active ingredients, and safety of the Canadian sea cucumbers in Chinese market were unknown, as well the differences compared with traditional Chinese sea cucumbers. Based on their processing method, the commercial Canadian sea cucumbers (CCSC) in Chinese food market were ascribed to different specifications, and their prices are different accordingly. In addition, for the same specification, the bigger size of sea cucumber, the higher price it sells. However, the difference of nutritional components was unknown. According to the focus points of consumers, the price, nutritional components, and safety (mainly discussed the safety in heavy metals) were studied. Besides that, the identification, characteristics, and size of CCSC were discussed.

In this research, different CCSC were collected and identified by their appearance, microscopic photographs, and DNA Barcoding method. The average contents of proteins, polysaccharide, amino acids, saponins, mineral elements, and heavy metals were determined to obtain information of their nutritional value and safety in heavy metals. In addition, for the CCSC of large‐scale sales, the relationships between their active components and sizes were investigated as well. The research provided consumers a reference for the varieties, nutritional value, and safety in heavy metals of Canadian sea cucumbers. Moreover, it was the foundation to consumers for the purchase of Canadian sea cucumber.

## 2 MATERIALS AND METHODS

### 2.1 Materials and sample collection

Five types of commercial dried products of Canadian sea cucumbers were randomly purchased from Arcitca Food Company and Guangzhou Qingping herbal medicine market in China, respectively. Each type was with similar size and randomly collected for 3 batches, at least 200 g per batch. Different size of CCSC in large‐scale circulation (Sample 2 and 3) was collected. All samples were labeled and stored at room temperature. The sample information was listed in Table 1. Canadian sea cucumber powder was prepared by crushing and sieving through 20 meshes. Marine Animals DNA Kit was purchased from Tiangen Biotech (Beijing, China). Ginsenoside Re (CAS: 52286‐59‐6) and Amino acids standard solution (16001) were obtained from National Institutes for Food and Drug Control (China) and National Institute of Metrology (China). Acetonitrile and methanol were of HPLC grade, and other analytical reagents were of AR grade.

**TABLE 1:** Information of commercial Canadian sea cucumbers

Sample number	Commercial name	Abbreviation	Commercial processing description	Amount/kilogram
1	Tubbish sea cucumber	S1	Cutting at both sides, viscera removal and light dried	50 ± 10
2	Cutting sea cucumber	S2	Cutting at one side, viscera removal	50 ± 10
3	Sea cucumber skin	S3	Cutting in the middle, viscera and inside wall removal	60 ± 10
4	Incompleted sea cucumber	S4	Cutting at one side, viscera removal and cutting into pieces	55 ± 10
5	Whole sea cucumber	S5	No intestinal tract removal	45 ± 10
6	Cutting sea cucumber	S6	Cutting at one side, viscera removal	75 ± 10
7	Cutting sea cucumber	S7	Cutting at one side, viscera removal	100 ± 10
8	Sea cucumber skin	S8	Cutting in the middle, viscera and inside wall removal	130 ± 10
9	Sea cucumber skin	S9	Cutting in the middle, viscera and inside wall removal	200 ± 10

### 2.2 Microscopic and DNA barcoding method

Ossicles were prepared according to the reported method (Liao, 1997) and photographed by a biological microscope (BK5000, OPTEC) to preliminarily identify the species. Subsequently, molecular identification of DNA barcoding based on 16S rRNA (16Sar: CGCCTGTTTATCAAAAACAT and 16Sbr: CTCCGGTTTGAACTCAGAT CA) mitochondrial sequences was used to further identify the species. Total DNA extraction, PCR amplification, DNA sequencing, and BLAST analysis at NCBI were performed as the reported method (Kerr et al., 2005; Zeng et al., 2018).

### 2.3 Morphological identification

The commercial Canadian sea cucumbers were observed, smelled, and tasted, and their appearance and structure were carefully observed to obtain the general characters. The appearance included color, size, and distribution of tube foot and characteristic of internal tendons and so on. Amount of powder was taken to observe its properties. Ten samples were randomly selected from each specification and soaked for 48 hr in 4°C. The body wall, abdomen, section, and internal tendon were observed. The weight before and after foaming was recorded, and the length, middle width, and thickness after soaking were measured. They were observed and measured as above method. Soaking degree is an important parameter that consumers pay attention to. It was calculated according to the following formula:

(1) Soaking degree=Weight after soaking/Dried body weight

### 2.4 Water content

Water content of Canadian sea cucumbers was determined by loss on drying method (Liu & Wang, 1995) and calculated according to the following formula:

(2) $$\text{Water Content}\;\left(\%\right)=\left(W_{1}‐W_{2}\right)/W_{1}\times 100$$

where *W*_1_ was weight of Canadian sea cucumber powder, and *W*_2_ was the powder weight after drying to a constant weight.

### 2.5 Nutritional compounds

#### 2.5.1 Proteins content

Kjeldahl method was used to determine proteins content of Canadian sea cucumbers using DigiPREP TKN Systems (FOSS‐8400, Switzerland) (Lynch & Barbano, 1999).

#### 2.5.2 Determination of free amino acids

Free amino acids analysis was determined by pre‐column derivatization‐high performance liquid chromatography (HPLC, 1260, Agilent). Sixteen free amino acids were determined by the comparison of their retention time and peak areas with the standards (Fernández‐Novales et al., 2019).

#### 2.5.3 Saponins content

The quantitative content of saponins was determined by HPLC, with ginsenoside Re as standard and obtained by comparing their retention time and peak areas with those of the standards (Kochan et al., 2018).

#### 2.5.4 Determination of polysaccharides

The polysaccharides were extracted and detected by phenol sulfuric acid method at 490 nm using fucose as the standard (Wu et al., 2018).

#### 2.5.5 Determination of mineral elements

Sample powders were digested in 65% nitric acid by microwave digestion system (MDS‐6G, SINEO), and the content of mineral elements was determined by inductively coupled plasma‐mass spectrometry (ICP‐MS, iCAP‐RQ, Thermo Fisher Scientific) using multielement standard solution for ICP analysis as the standard (0.1–10,000 ng/ml, GNM‐M301629‐2013) (Fragni et al., 2018; Jia et al., 2019).

### 2.6 Determination of heavy metal

Sample preparation and detection method were the same as Section 2.5.5.

### 2.7 Statistical analysis

Statistical analyses were performed with SPSS 20.0 (IBM Corporation) of at least three independent experiments. All quantitative data were expressed as mean ± *SD*.

## 3 RESULTS AND DISCUSSION

### 3.1 Species identification and introduction

**FIGURE 1:**
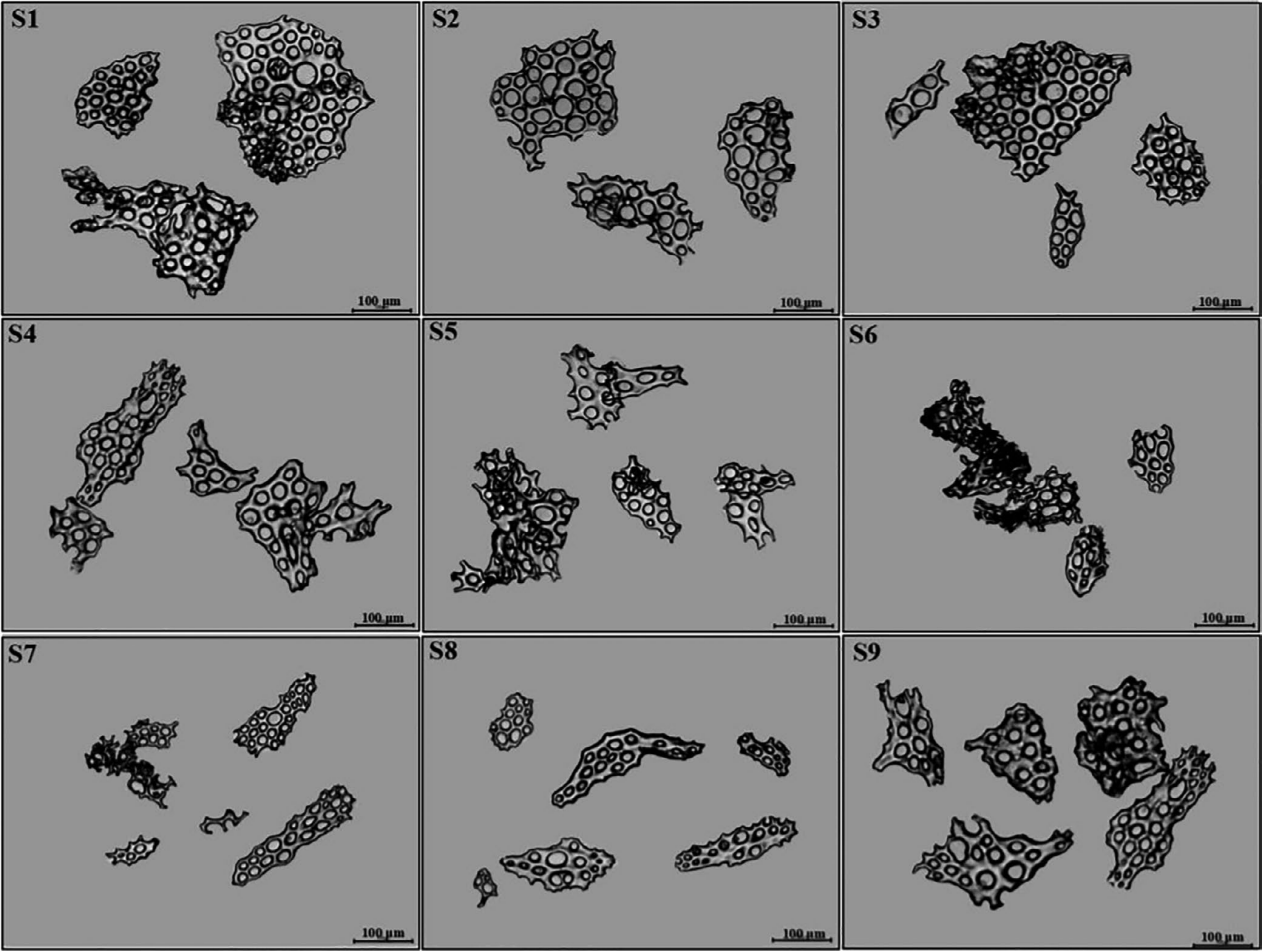
Microscopic characters of commercial Canadian sea cucumbers

The shape and size of ossicles in the epidermis of the sea cucumber vary from species, which is an important basis for the classification of sea cucumbers (Liao, 1997). Microscopic identification results in Figure 1 indicated that the ossicles of the body wall were perforated plates with different sizes and the ossicles shape was triangular, quadrangular, or subcircular with ragged edge, which fitted the microscopic character of *Cucumaria frondosa*. The surface of the perforated plates was either smooth or with projections. Molecular identification of DNA barcoding based on 16S rRNA mitochondrial sequences showed that the matching degree of the studied CCSC with *Cucumaria frondosa* was 99.4%–100% using Blast analysis, and the e‐value was zero. The landing number of target sequence was KF479389.1 in GeneBank. In summary, microscopic and molecular identification showed that the CCSC were the dried body of *Cucumaria frondosa* from Echinodermata: Holothuroide, Dendrochirotida, Cucumariidae, Cucumaria. These microscopic and molecular methods could be further used to identify the truth of *Cucumaria frondosa*.

**FIGURE 2:**
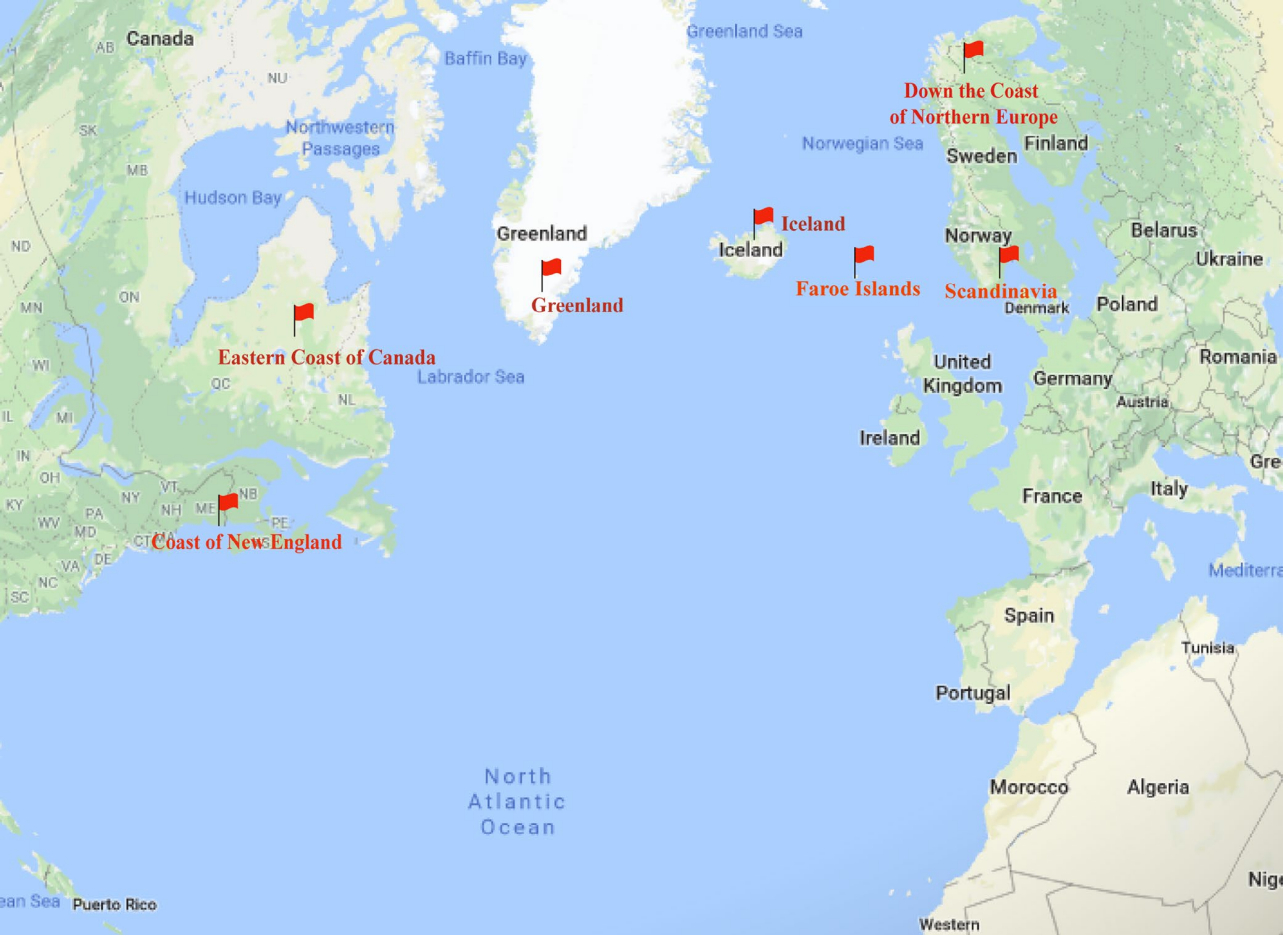
The majority distribution in North Atlantic Ocean of *Cucumaria frondosa*. The map was from www.google.cn/maps

There are many alias names of *Cucumaria frondosa*, such as Orange‐footed sea cucumber, Northern sea cucumber, Atlantic sea cucumber, Canadian sea cucumber, or Icelandic sea cucumber (Nelson et al., 2012). It mainly geographically distributed at depths of 30–100 meters in the North Atlantic Ocean (Zhao, Lin, & Liu, 2016; Sun, Hamel, & Mercier, 2018), such as the coast of New England (USA), the eastern coast of Canada, Iceland and Greenland, down the coast of northern Europe and Scandinavia, as well as in the Faroe Islands (Figure 2). In addition, it could also be found in Barents Sea of Russia. The fishery of *Cucumaria frondosa* began in Maine (USA) in the 1980s (So, Uthicke, Hamel, & Mercier, 2011). Due to the high content of autolytic enzymes, sea cucumber needs to be treated immediately after fishing, so that it can be transported for sale (Fu, Xue, Miao, & Li, 2005). In Canada, most of *Cucumaria frondosa* are exported, and the major market is Asian, especially China. *Cucumaria frondosa* were mostly consumed as bêche‐de‐mer (trepang or dried body), and a few as canned (undried) product. It could be used commercially for the preparation of delicious food and traditional medicinal products. Currently, the prices of the dried body of CCSC were 200–3,000 RMB per kilogram. The bought dried body of CCSC could cook soup, fry and so on after soaking, which tasted delicious.

### 3.2 Characteristics and character differences of CCSC

**FIGURE 3:**
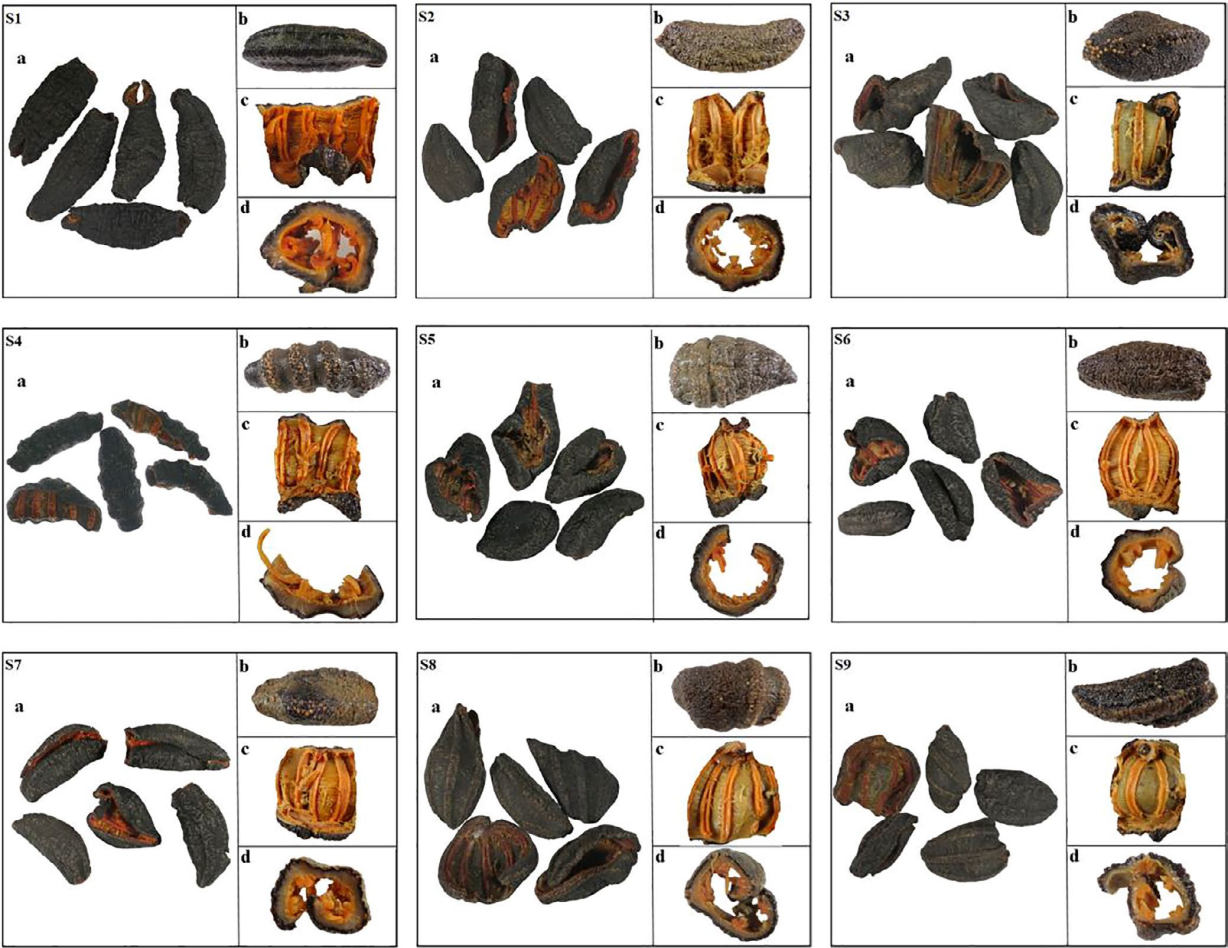
Characteristics of sea cucumber from Canada (a. overall; b. body wall and tube foot after soaking; c. inner wall and rib after soaking; d. cross‐section after soaking.)

There were similarities in general characters among different commercial *Cucumaria frondosa*. As shown in Figure 3, their bodies were cylindrical, slightly curved dorsally, tapered gently at both ends, smelled fishy, and tasted salty. The body color was light or dark black, and the body has a slightly grainy surface with visible five rows of yellowish podia. The abdomen and five internal tendons are unique orange or golden. The inner wall was also orange or golden, occasionally contained residual intestinal tract and dendritic tentacles. After soaking, the body surface was sticky and slippery. The general character description could be a foundation for consumers to select Canadian sea cucumber. The average of soaking degree and water content in CCSC, which consumers were paid great attention to, was 2.8 ± 0.3 and 0.46 ± 0.09%, respectively.

**FIGURE 4:**
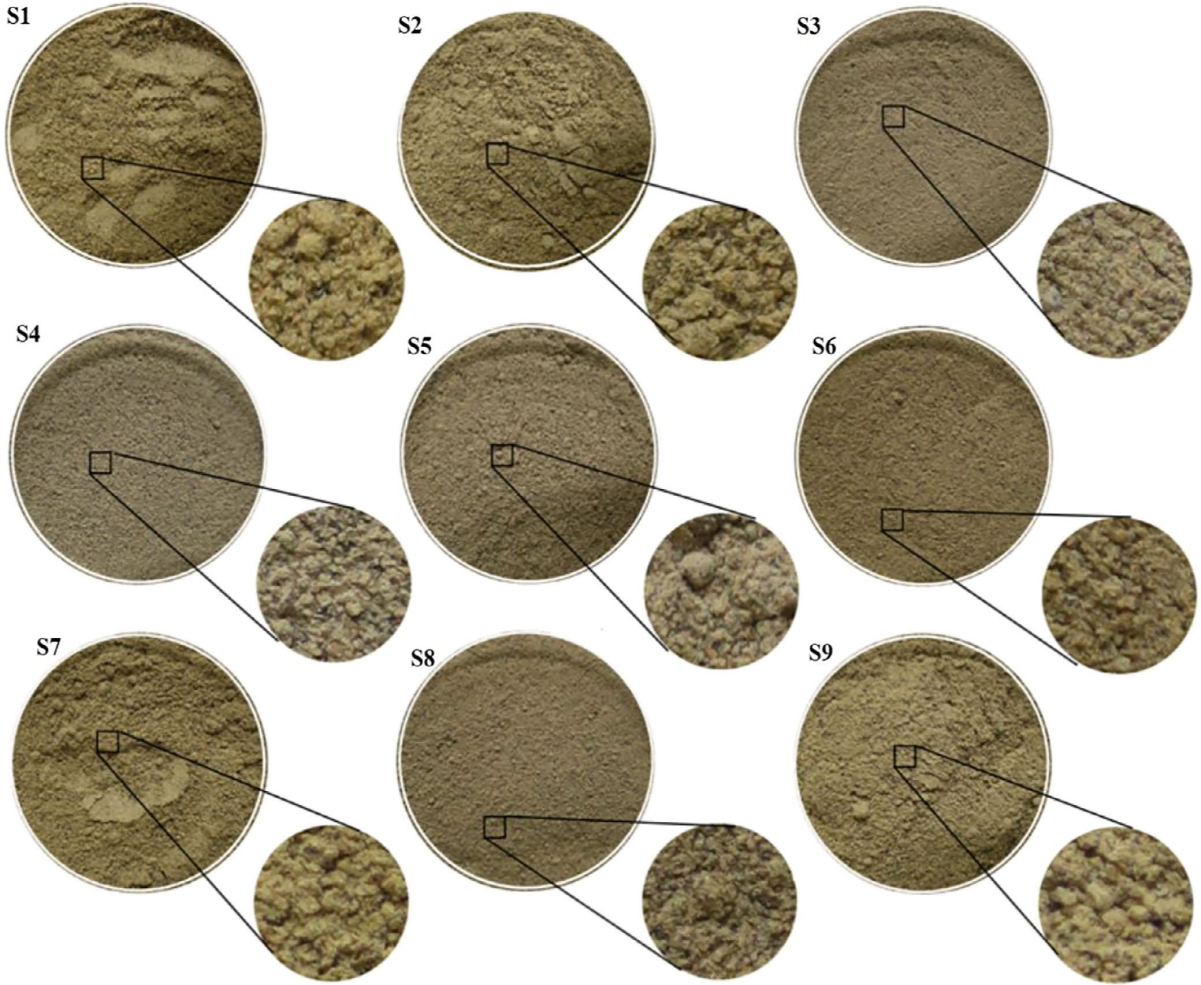
Powder characteristics of Canadian sea cucumber

The characters of different commercial *Cucumaria frondosa* were shown in Table 2. Among them, the body of S1 was relatively intact and fullness, the color of the inner wall and tendon was bright dark orange. And S1 had the highest soaking degree (3.1 ± 0.0) and water content (0.61 ± 0.03%). Moreover, the price of S1 was the highest. Because of scraped out part section of inner wall and tendon, S3 was called “Sea cucumber skin,” whose body wall was the thinnest and the color was the lightest. Market research findings showed that S4 was cut into pieces, which was incomplete body and had most obvious five rows of yellowish podia. S5 had the largest body with intact mouth and anus, as well as the thickest body wall. Furthermore, S5 had most complete structure, so commonly called as “Whole sea cucumber,” in which intestinal tract and dendritic tentacles could be observed clearly. The powders of all samples were brown and slightly fishy and there was no notable difference found among them (Figure 4). Character differences between different size in “Cutting sea cucumber” and “Sea cucumber skin” indicated that the thickness, length, and girth were proportional to size, but not the soaking degree and water content.

**TABLE 2:** Characteristics parameters of different commercial *Cucumaria frondosa*

Sample No.	S1	S2	S3	S4	S5	S6	S7	S8	S9
Body color	Dark black	Black	Light black	Black	Black	Black	Black	Light black	Light black
Rib color	Dark orange	Light orange	Light orange	Light orange	Light orange	Light orange	Light orange	Light orange	Light orange
Inner wall color	Dark orange	Light orange	Yellow‐green	Light orange	Light orange	Light orange	Light orange	Yellow‐green	Yellow‐green
Soaking degree	3.1 ± 0.3	2.5 ± 0.1	2.9 ± 0.2	2.9 ± 0.2	2.5 ± 0.1	2.5 ± 0.03	2.5 ± 0.03	2.9 ± 0.2	2.9 ± 0.2
Length after soaking (cm)	10.7 ± 1.1	9.8 ± 0.3	8.7 ± 0.2	8.0 ± 0.8	10.1 ± 1.2	8.0 ± 0.5	7.2 ± 0.6	7.4 ± 0.6	6.7 ± 0.6
Girth after soaking (cm)	10.2 ± 1.2	8.9 ± 0.5	8.8 ± 0.2	7.6 ± 1.7	10.5 ± 0.7	8.6 ± 0.5	7.3 ± 0.4	7.2 ± 0.3	7.1 ± 0.3
Thickness not contained podia after soaking (cm)	0.41 ± 0.04	0.48 ± 0.09	0.31 ± 0.09	0.44 ± 0.11	0.47 ± 0.09	0.35 ± 0.06	0.30 ± 0.02	0.28 ± 0.04	0.26 ± 0.04
Thickness contained podia after soaking (cm)	0.75 ± 0.10	0.88 ± 0.03	0.72 ± 0.20	0.72 ± 0.06	1.15 ± 0.14	0.83 ± 0.12	0.65 ± 0.10	0.66 ± 0.11	0.61 ± 0.09
Water content (%)	0.61 ± 0.03	0.38 ± 0.04	0.45 ± 0.03	0.43 ± 0.01	0.38 ± 0.04	0.41 ± 0.02	0.41 ± 0.01	0.45 ± 0.01	0.47 ± 0.03

### 3.3 Content of active components

#### 3.3.1 Proteins content

**FIGURE 5:**
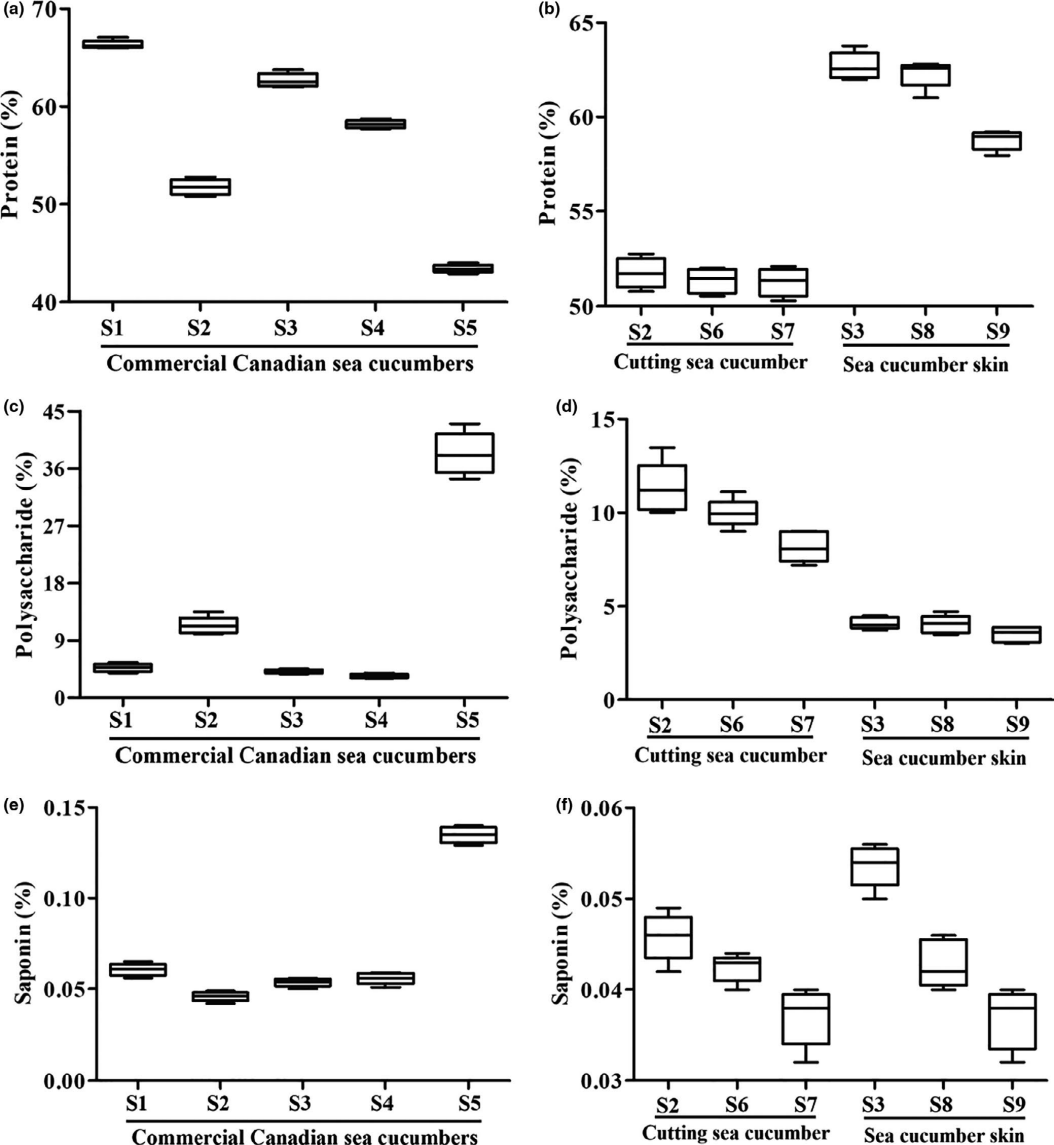
The content of protein (a), polysaccharide (c), saponin (e) from different commercial *Cucumaria frondosa* and content of protein (b), polysaccharide (d), saponin (f) from different size of *Cucumaria frondosa*

Protein is the main component in dry sea cucumber (Xiong et al., 2020), which may be the prime component in common sense of consumers. As shown in Figure 5a, the average protein content of *Cucumaria frondosa* was 56.4 ± 9.1% of the dried weight, which exceeded total ingredients of 50%. The protein content in commercial *Cucumaria frondosa* was S1 (66.4 ± 0.6%), S2 (51.4 ± 0.8%), S3 (62.5 ± 0.5%), S4 (58.0 ± 0.3%), and S5 (43.4 ± 0.6%), respectively. Furthermore, the protein content was positively correlated with size in different size group (Figure 5b).

#### 3.3.2 Content of polysaccharides

The natural and unique structural polysaccharides from sea cucumber are found to be multi‐effective and relatively nontoxic substances, and have been attracted more attention due to various pharmaceutical and nutritional functions (Xiong et al., 2020). The average content of polysaccharides was 12.2 ± 14.7% of the dried weight. The polysaccharide content in Figure 5c was S1 (4.6 ± 0.6%), S2 (10.9 ± 0.8%), S3 (4.2 ± 0.3%), S4 (3.5 ± 0.4%), and S5 (38.1 ± 1.7%), respectively. The highest in polysaccharides contents in S5 might be related to the unremoved intestinal tract (Yuan et al., 2010). The content of polysaccharides in S5, after removing intestinal tract, was 7.5 ± 0.7%. The results about different size of *Cucumaria frondosa* demonstrated that the polysaccharides content was in size‐dependently (Figure 5d).

#### 3.3.3 Saponin content

Saponins are characteristic metabolites of sea cucumbers (Xiao, Shao, Zhu, & Yu, 2019). Triterpene, a major active saponin in *Cucumaria frondosa*, possessed strong biological activity (Aminin et al., 2008). As shown in Figure 5e, the average saponin content of *Cucumaria frondosa* (0.070 ± 0.036%) was almost close to 1‰ of total ingredients. The saponin content in CCSC was S1 (0.061 ± 0.005%), S2 (0.046 ± 0.004%), S3 (0.053 ± 0.003%), S4 (0.055 ± 0.004%), and S5 (0.13 ± 0.01%), respectively. Similar to polysaccharides, the saponin content was positively correlated with size which was presented in Figure 5f.

#### 3.3.4 The content of free amino acids

**FIGURE 6:**
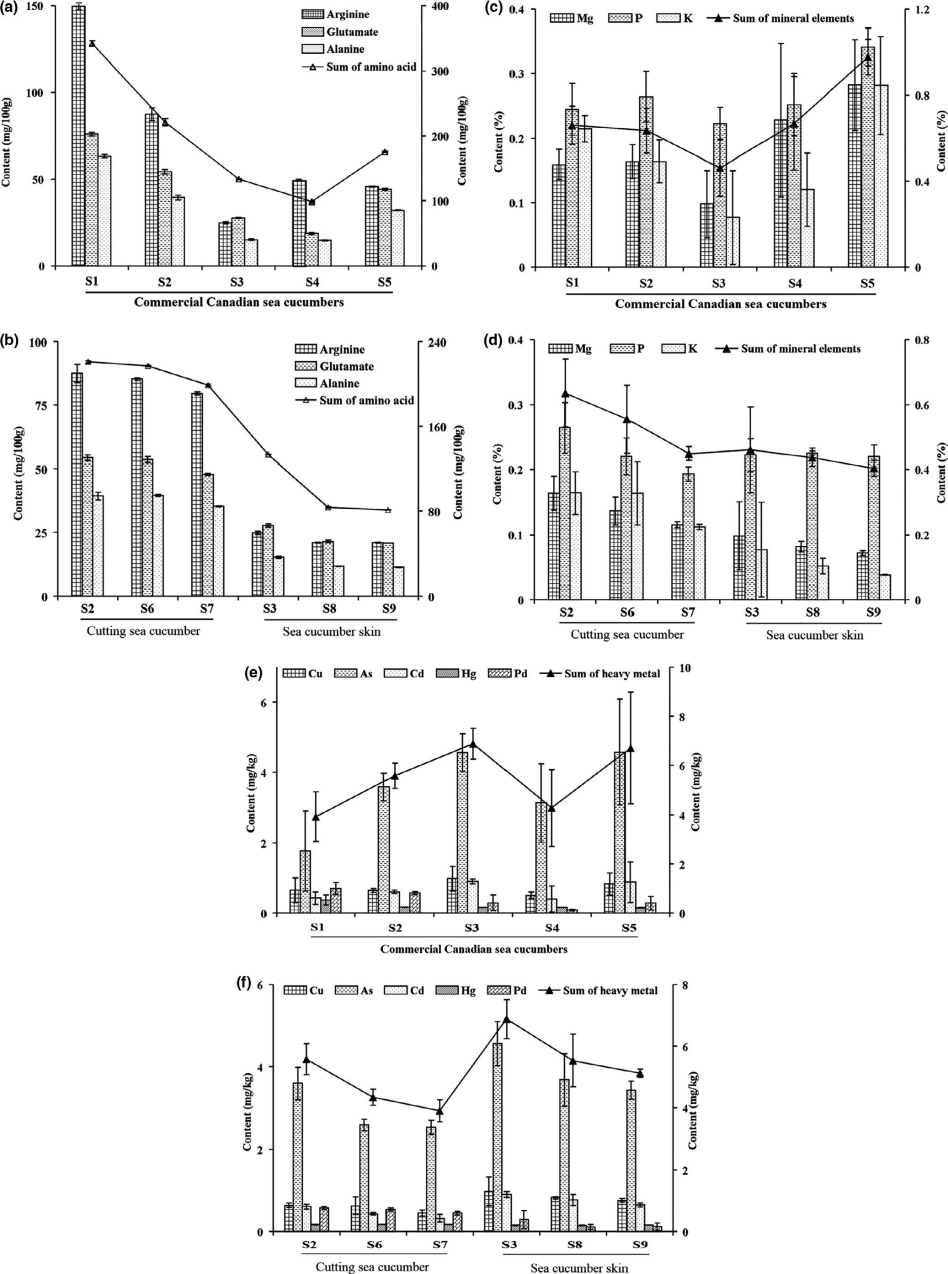
The content of amino acid (a), major mineral elements (c) and heavy metal (e) from different commercial *Cucumaria frondosa* and content of amino acid (b), major mineral elements (d) and heavy metal (f) of different size. The content of column was the coordinate axis on the left, and line diagram was the right

Except protein, amino acids are comparatively important component in common sense of consumers. Sixteen kinds of free amino acids were all detected, in which arginine, glutamic acid, and alanine were the main components (Table 3). The average content of total sixteen free amino acids of CCSC was 0.18 ± 0.10%, major containing of arginine (70.1 ± 50.0 mg/100 g), glutamate (42.6 ± 23.9 mg/100 g), and alanine (32.2 ± 21.0 mg/100 g) (Figure 6a). Total amino acids and sixteen kinds of amino acids were almost all in a size‐dependent manner (Figure 6b and Table 3).

**TABLE 3:** The content of amino acid from *Cucumaria frondosa*

Content (mg/100 g)	S1	S2	S3	S4	S5	S6	S7	S8	S9
Aspartic acid	1.7 ± 0.01	2.2 ± 0.01	4.5 ± 0.1	1.0 ± 0.02	1.6 ± 0.03	2.1 ± 0.1	2.0 ± 0.03	1.8 ± 0.02	1.6 ± 0.02
Glutamate	76.0 ± 1.1	54.2 ± 0.1.1	27.7 ± 0.4	18.8 ± 0.6	44.0 ± 0.5	53.5 ± 0.4	47.8 ± 0.9	21.4 ± 0.6	20.7 ± 0.1
Serine	4.3 ± 0.1	4.5 ± 0.0.02	4.2 ± 0.02	1.7 ± 0.05	3.6 ± 0.1	4.4 ± 0.1	4.1 ± 0.03	3.4 ± 0.1	3.3 ± 0.02
Glycine	5.0 ± 0.04	4.4 ± 0.04	11.3 ± 0.1	1.7 ± 0.02	18.5 ± 0.3	4.4 ± 0.1	4.0 ± 0.05	3.3 ± 0.01	3.0 ± 0.00
Threonine	5.2 ± 0.1	4.0 ± 0.00	3.7 ± 0.02	1.2 ± 0.1	3.8 ± 0.02	4.1 ± 0.1	3.7 ± 0.1	2.2 ± 0.04	2.1 ± 0.01
Histidine	1.2 ± 0.02	0.91 ± 0.1	0.9 ± 0.02	0.3 ± 0.01	1.0 ± 0.04	0.70 ± 0.02	0.66 ± 0.02	0.5 ± 0.01	0.62 ± 0.00
Alanine	63.3 ± 1.0	39.3 ± 0.1.5	15.2 ± 0.4	14.8 ± 0.3	32.1 ± 0.1	39.6 ± 0.4	35.3 ± 0.2	11.6 ± 0.2	11.2 ± 0.05
Arginine	149.5 ± 1.9	87.4 ± 3.6	24.8 ± 0.6	49.4 ± 0.6	45.5 ± 0.4	85.2 ± 0.5	79.4 ± 0.6	20.8 ± 0.2	20.9 ± 0.1
Tyrosine	3.5 ± 0.04	2.6 ± 0.1	5.3 ± 0.02	1.3 ± 0.1	2.7 ± 0.03	2.6 ± 0.1	2.4 ± 0.04	1.8 ± 0.04	2.2 ± 0.03
Valine	4.3 ± 0.1	2.8 ± 0.05	7.3 ± 0.2	0.80 ± 0.02	1.8 ± 0.02	2.8 ± 0.03	2.5 ± 0.03	3.3 ± 0.04	2.7 ± 0.02
Methionine	0.31 ± 0.03	0.40 ± 0.1	0.5 ± 0.01	0.13 ± 0.00	0.14 ± 0.00	0.40 ± 0.00	0.41 ± 0.03	0.15 ± 0.02	0.11 ± 0.02
Phenylalanine	3.9 ± 0.1	3.5 ± 0.1	6.0 ± 0.1	1.0 ± 0.00	3.4 ± 0.03	4.0 ± 0.4	4.0 ± 0.1	3.3 ± 0.02	3.7 ± 0.1
Isoleucine	4.3 ± 0.1	2.1 ± 0.03	4.1 ± 0.1	0.97 ± 0.05	1.8 ± 0.02	1.9 ± 0.2	1.6 ± 0.01	1.9 ± 0.02	1.7 ± 0.03
Leucine	4.6 ± 0.00	3.5 ± 0.1	6.8 ± 0.04	0.95 ± 0.03	2.6 ± 0.03	3.2 ± 0.2	3.0 ± 0.05	2.9 ± 0.03	2.9 ± 0.1
Lysine	9.2 ± 0.05	5.2 ± 0.02	4.3 ± 0.03	2.8 ± 0.02	8.7 ± 0.3	5.0 ± 0.2	4.5 ± 0.04	2.0 ± 0.05	1.8 ± 0.02
Proline	5.0 ± 0.04	3.6 ± 0.05	6.3 ± 0.03	1.5 ± 0.02	4.0 ± 0.04	3.0 ± 0.5	3.5 ± 0.01	3.1 ± 0.03	2.6 ± 0.05
Totality	341.3 ± 4.1	220.6 ± 5.7	133.1 ± 0.7	98.2 ± 1.6	175.3 ± 1.3	216.5 ± 1.2	198.7 ± 2.0	83.5 ± 1.1	81.1 ± 0.3

#### 3.3.5 The content of mineral elements

Mineral elements are supplements which human need and consumers care. The abbreviations of mineral elements were shown in Table 4. P (0.26 ± 0.05%), Mg (0.19 ± 0.07%), and K (0.17 ± 0.08%) were the major elements in *Cucumaria frondosa*, presented in Table 4 and Figure 6c. Followed, Ca, Fe, Zn, Sr, and Al were higher than the other remaining elements. The average content of mineral elements was 0.68 ± 0.19%. The mineral elements contents are also positively correlated with the size of *Cucumaria frondosa* (Figure 6d).

**TABLE 4:** The content of mineral element from *Cucumaria frondosa*

Content (mg/kg)		S1	S2	S3	S4	S5	S6	S7	S8	S9
Lithium	Li	0.07 ± 0.01	0.05 ± 0.02	0.02 ± 0.02	0.07 ± 0.05	0.08 ± 0.03	0.04 ± 0.01	0.02 ± 0.00	0.02 ± 0.01	0.01 ± 0.00
Beryllium	Be	0.04 ± 0.04	0.01 ± 0.00	0.01 ± 0.00	0.01 ± 0.00	0.01 ± 0.00	0.01 ± 0.00	0.01 ± 0. 00	0.01 ± 0.00	0.01 ± 0.00
Boron	B	2.94 ± 0.27	4.10 ± 0.73	1.81 ± 0.96	4.17 ± 2.11	4.98 ± 0.83	2.34 ± 0.49	1.93 ± 0.23	1.75 ± 0.13	1.33 ± 0.30
Magnesium	Mg	1589.17 ± 232.00	1633.37 ± 261.86	978.32 ± 521.39	2,278.70 ± 1,188.59	2,819.96 ± 703.69	1,363.21 ± 216.00	1,154.04 ± 45.47	817.91 ± 82.74	717.23 ± 37.47
Aluminum	Al	13.80 ± 4.74	/	15.85 ± 0.23	7.80 ± 7.64	6.32 ± 4.33	/	/	21.53 ± 2.45	19.40 ± 5.66
Phosphorus	P	2,449.61 ± 400.66	2,642.96 ± 389.42	2,225.90 ± 249.99	2,518.23 ± 482.73	3,411.92 ± 292.32	2,201.55 ± 283.72	1931.55 ± 109.07	2,257.11 ± 27.71	2,201.94 ± 174.26
Kalium	K	2,141.76 ± 206.56	1638.88 ± 328.97	769.68 ± 727.84	1,202.14 ± 560.82	2,817.27 ± 760.19	1634.93 ± 485.22	1,116.17 ± 41.72	516.37 ± 123.38	378.17 ± 6.31
Calcium	Ca	306.89 ± 39.93	312.75 ± 53.22	474.30 ± 147.82	531.49 ± 64.77	550.70 ± 126.98	254.31 ± 51.22	208.84 ± 7.86	595.84 ± 43.65	553.68 ± 21.17
Vanadium	V	0.21 ± 0.04	0.17 ± 0.02	0.23 ± 0.05	0.28 ± 0.04	0.28 ± 0.03	0.15 ± 0.02	0.12 ± 0.01	0.34 ± 0.01	0.32 ± 0.06
Manganese	Mn	1.07 ± 0.03	1.89 ± 0.29	1.40 ± 0.36	2.12 ± 0.62	3.24 ± 0.29	1.07 ± 0.25	1.31 ± 0.03	1.44 ± 0.17	1.28 ± 0.08
Ferrum	Fe	29.35 ± 4.54	26.30 ± 5.49	43.44 ± 9.90	31.67 ± 11.64	32.14 ± 3.18	22.67 ± 6.32	16.16 ± 0.75	50.46 ± 2.94	55.49 ± 4.43
Cobalt	Co	0.11 ± 0.04	0.07 ± 0.01	0.07 ± 0.01	0.08 ± 0.00	0.080 ± 0.01	0.06 ± 0.01	0.05 ± 0.00	0.08 ± 0.01	0.07 ± 0.01
Nickel	Ni	0.49 ± 0.10	0.35 ± 0.04	0.52 ± 0.13	0.35 ± 0.05	0.73 ± 0.45	0.25 ± 0.08	0.23 ± 0.09	0.48 ± 0.11	0.41 ± 0.09
Cuprum	Cu	0.65 ± 0.35	0.64 ± 0.06	0.98 ± 0.35	0.51 ± 0.09	0.82 ± 0.31	0.63 ± 0.22	0.45 ± 0.07	0.83 ± 0.03	0.76 ± 0.04
Zinc	Zn	30.59 ± 3.09	38.57 ± 3.36	47.00 ± 8.86	38.29 ± 7.98	35.12 ± 4.61	30.00 ± 0.30	30.33 ± 1.46	53.20 ± 1.32	54.50 ± 5.67
Gallium	Ga	0.08 ± 0.09	0.01 ± 0.00	0.01 ± 0.00	0.01 ± 0.00	0.01 ± 0.00	0.01 ± 0.00	0.004 ± 0.001	0.01 ± 0.00	0.01 ± 0.00
Arsenic	As	1.77 ± 1.15	3.59 ± 0.39	4.56 ± 0.54	3.13 ± 1.12	4.58 ± 1.51	2.58 ± 0.14	2.52 ± 0.17	3.68 ± 0.64	3.34 ± 0.22
Selenium	Se	2.21 ± 0.39	1.47 ± 0.13	1.76 ± 0.27	1.75 ± 0.18	2.62 ± 0.34	1.71 ± 0.64	1.11 ± 0.03	2.14 ± 0.11	2.10 ± 0.07
Rubidium	Rb	0.76 ± 0.87	0.15 ± 0.03	0.08 ± 0.07	0.11 ± 0.04	0.26 ± 0.07	0.15 ± 0.04	0.10 ± 0.01	0.06 ± 0.01	0.05 ± 0.00
Strontium	Sr	30.17 ± 2.96	37.33 ± 4.18	40.55 ± 4.09	61.61 ± 12.94	79.59 ± 6.57	29.04 ± 0.79	29.17 ± 0.70	47.79 ± 4.31	43.29 ± 1.32
Molybdenum	Mo	0.21 ± 0.08	0.31 ± 0.03	0.25 ± 0.04	0.55 ± 0.20	0.47 ± 0.20	0. 17 ± 0.05	0. 24 ± 0.02	0.28 ± 0.03	0.27 ± 0.03
Cadmium	Cd	0.42 ± 0.18	0.61 ± 0.05	0.90 ± 0.07	0.40 ± 0.37	0.88 ± 0.58	0.43 ± 0.03	0.32 ± 0.09	0.77 ± 0.13	0.65 ± 0.05
Antimony	Sb	0.11 ± 0.14	0.03 ± 0.01	0.03 ± 0.01	0.03 ± 0.01	0.03 ± 0.01	0.03 ± 0.01	0.02 ± 0.00	0.04 ± 0.03	0.02 ± 0.00
Barium	Ba	1.54 ± 0.14	1.09 ± 0.23	1.14 ± 0.16	1.18 ± 0.25	1.50 ± 0.35	1.21 ± 0.40	0.73 ± 0.16	1.39 ± 0.29	1.07 ± 0.18
Mercury	Hg	0.37 ± 0.15	0.17 ± 0.01	0.16 ± 0.00	0.15 ± 0.00	0.15 ± 0.00	0.17 ± 0.00	0.17 ± 0.01	0.15 ± 0.00	0.16 ± 0.00
Plumbum	Pb	0.70 ± 0.17	0.57 ± 0.04	0.29 ± 0.22	0.08 ± 0.02	0.28 ± 0.19	0.53 ± 0.04	0.45 ± 0.04	0.11 ± 0.06	0.12 ± 0.09
Bismuth	Bi	0.12 ± 0.15	0.02 ± 0.00	0.01 ± 0.01	0.01 ± 0.00	0.10 ± 0.00	0.01 ± 0.00	0.02 ± 0.00	0.01 ± 0.00	0.01 ± 0.00
Sum (%)		0.66 ± 0.09	0.63 ± 0.10	0.46 ± 0.13	0.67 ± 0.22	0.98 ± 0.08	0.56 ± 0.10	0.45 ± 0.02	0.44 ± 0.03	0.40 ± 0.02
Sum of heavy metal (mg/kg)		3.9 ± 1.0	5.6 ± 0.5	6.9 ± 0.6	4.3 ± 1.6	6.7 ± 2.3	4.3 ± 0.3	3.9 ± 0.4	5.5 ± 0.9	5.1 ± 0.1

Note

Symbol “/” meaned no detection in sample.

### 3.4 The safety of heavy metals in *Cucumaria frondosa*

Food safety in chemistry mostly indicates heavy metals, toxin, pesticide, radiation and so on. The areas Canadian sea cucumber distributed was unfrequented and had no agriculture. Aquatic animals could enrich heavy metals in water, which might lead to heavy metals exceeding the standard. Therefore, we tested the heavy metals content in *Cucumaria frondosa*. Cd, Hg, Pd, As, and Cu were generally regarded as the main heavy metals pollution (You et al., 2016). Average total amount of heavy metal was within the safety limitation (5.5 ± 1.4 mg/kg < 20 mg/kg). Furthermore, the content of heavy metal elements was S1 (3.9 ± 1.0 mg/kg), S2 (5.6 ± 0.5 mg/kg), S3 (6.9 ± 0.6 mg/kg), S4 (4.3 ± 1.6 mg/kg), and S5 (6.7 ± 2.3 mg/kg), indicating CCSC were all safety in heavy metals to consumers (Figure 6e). The size of *Cucumaria frondosa* was positively correlated with heavy metal (Figure 6f), and same as in mineral elements.

## 4 CONCLUSIONS

**FIGURE 7:**
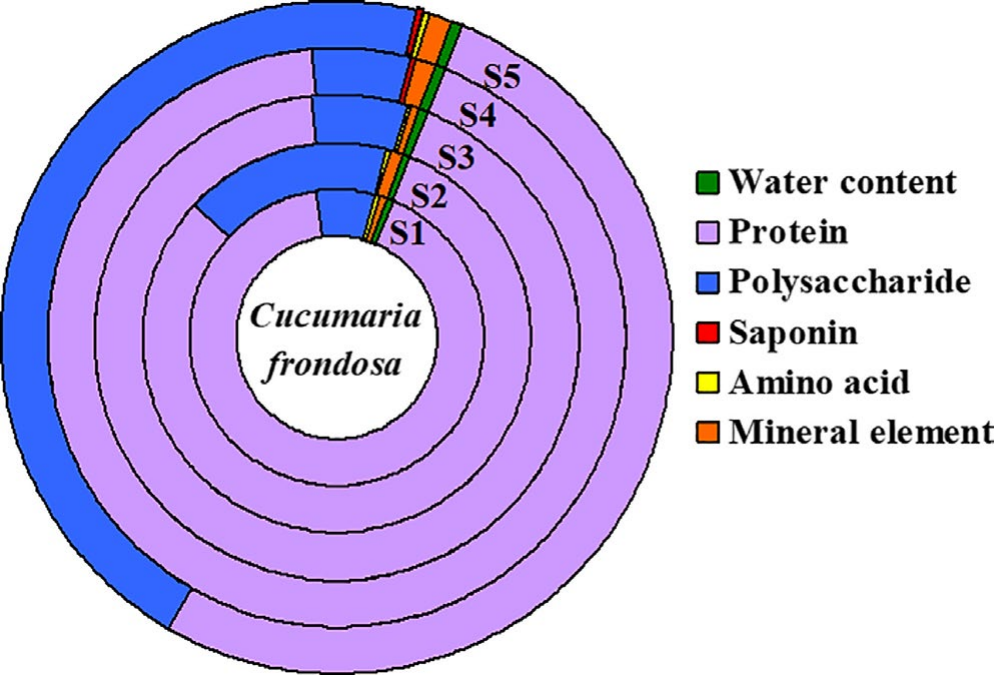
The average content of active ingredients in different commercial *Cucumaria frondosa*

According to the issues which consumers paid attention to, abundant information of CCSC was systemically investigated, such as nutritional components, price, safety, taste, locality, size and specification, variety, and soaking degree. Firstly, in order to clarify the classification, CCSC were identified by microscopic and molecular identification as dried body wall from *Cucumaria frondosa*. The abdomen and five internal tendons of *Cucumaria frondosa* are special orange or golden, so they are also called Orange‐footed sea cucumber. Studies on active components (summed in Figure 7) indicated that protein and polysaccharide were the principal component. Saponins were important and effective active compound in *Cucumaria frondosa*, which possessed strong biological activity. A variety of free amino acids (182.6 ± 104.7 mg/100 g) was detected in CCSC, among which arginine, glutamic acid, and alanine were the main components. Phosphorus (P, 0.26 ± 0.05%), magnesium (Mg, 0.19 ± 0.07%), and kalium (K, 0.17 ± 0.08%) were the major elements. The heavy metal content was within the safety limitation, indicating the safety in heavy metal of CCSC. The difference between content of active components might be related to the processing methods. The size and thickness of *Cucumaria frondosa* in same market specification were positively correlated with active ingredients content.

*Cucumaria frondosa* is a marine animal, which is wild, high yield, and low price. The average contents of protein, polysaccharide, saponins, amino acid, mineral elements, and heavy metal were 56.4 ± 9.1%, 12.2 ± 14.7%, 0.070 ± 0.036%, 0.18 ± 0.10%, 0.68 ± 0.19%, and 5.5 ± 1.4 mg/kg, respectively. The character, protein content, and water content of CCSC were all matched the first grade description of *Stichopus Japonicus* in Chinese standards of GB/T 34747‐2017. Compared with other sea cucumbers, the composition and content were not much difference to those of other sea cucumbers (Chen, Xue, Tang, Yu, & Chai, 2011), which was shown in Table 5. The overall findings also suggested that the quality of *Cucumaria frondosa* was varied according to the size. Among them, S2 and S3 were mass circulation in the market and the price was 200–700 RMB per kilogram, while S1 was 1,500–4,000. The results enriched the information of nutritional value and safety in heavy metal of CCSC. If the wild *Cucumaria frondosa* was to be used as food in a large scale, we do not need to worry about the lack of resources, since it could be captive cultured (Nelson et al., 2012).

**TABLE 5:** The content of nutritional compounds compared in different sea cucumbers

Species name	Content of polysaccharide	Content of protein	Content of amino acids	Reference
*Cucumaria frondosa*	12.2%	56.4%	0.18% (free)	/
*Apostichopus japonicus*	64.21% or 0.25%–0.28% (wet weight)	4.1%–4.7% (wet weight)	31.9%–33.5% (hydrolysis)	Jiang, Dong, Gao, Wang, and Tian (2013)
*Stichopus japonicus*	7.4%–8.4%	43.3%–51.5%	37.4%–46.0% (hydrolysis)	Chen et al. (2011)
*Stichopus tremulus*	7.0%	/	/	Chen et al. (2011)
*Holothuria vagabunda*	6.3%	/	/	Chen et al. (2011)
*Isostichopus badionotus*	9.9%	/	/	Chen et al. (2011)
*Pearsonothuria graeffei*	11.0%	/	/	Chen et al. (2011)
*Athyonidium chilensis*	4.2%	/	/	Matsuhiro, Osorio‐Román, and Torres (2012)
*Stichopus herrmanni*	/	47.0%	34.7% (hydrolysis)	Wen, Hu, and Fan (2010)
*Thelenota ananas*	/	55.2%	42.3% (hydrolysis)	Wen et al. (2010)
*Holothuria fuscogilva*	/	57.8%	53.4% (hydrolysis)	Wen et al. (2010)
*Holothuria fuscopunctata*	/	50.1%	49.4% (hydrolysis)	Wen et al. (2010)
*Actinopyga mauritiana*	/	63.3%	54.1% (hydrolysis)	Wen et al. (2010)
*Bohadschia argus*	/	62.1%	47.3% (hydrolysis)	Wen et al., 2010)
*Holothuria polii*	/	8.8% (wet weight)	/	Aydın, Sevgili, Tufan, Emre, and Köse (2011)
*Holothuria mammata*	/	7.9% (wet weight)	/	Aydın et al. (2011)

Note

Symbol “/” meaned no detection in sample.

## Supporting information

Figure S1
