# Supplementary material for: Consumers' attention on identification, nutritional compounds, and safety in heavy metals of Canadian sea cucumber in Chinese food market
Source: Food Sci Nutr. 2020 Sep 13;8(11):5962–75. doi: 10.1002/fsn3.1882 (PMC7684582; doi:10.1002/fsn3.1882)

**Supplementary Fig. 1** The results of market research reports which consumers paid more attention to.


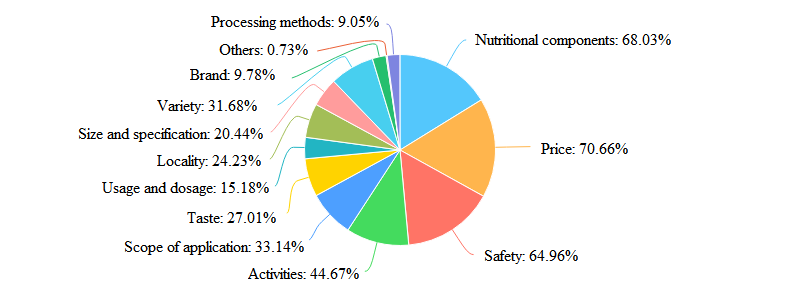

Supplement: Supplementary file 1 — Figure S1 [file FSN3-8-5962-s001.doc]
